# Low energy electron imaging of domains and domain walls in magnesium-doped lithium niobate

**DOI:** 10.1038/srep33098

**Published:** 2016-09-09

**Authors:** G. F. Nataf, P. Grysan, M. Guennou, J. Kreisel, D. Martinotti, C. L. Rountree, C. Mathieu, N. Barrett

**Affiliations:** 1 SPEC, CEA, CNRS, Université Paris-Saclay, CEA Saclay, 91191 Gif-sur-Yvette Cedex, France; 2 Materials Research and Technology Department, Luxembourg Institute of Science and Technology, 41 rue du Brill, 4422 Belvaux, Luxembourg; 3 Physics and Materials Science Research Unit, University of Luxembourg, 41 rue du Brill, 4422 Belvaux, Luxembourg

## Abstract

The understanding of domain structures, specifically domain walls, currently attracts a significant attention in the field of (multi)-ferroic materials. In this article, we analyze contrast formation in full field electron microscopy applied to domains and domain walls in the uniaxial ferroelectric lithium niobate, which presents a large 3.8 eV band gap and for which conductive domain walls have been reported. We show that the transition from Mirror Electron Microscopy (MEM – electrons reflected) to Low Energy Electron Microscopy (LEEM – electrons backscattered) gives rise to a robust contrast between domains with upwards (P_up_) and downwards (P_down_) polarization, and provides a measure of the difference in surface potential between the domains. We demonstrate that out-of-focus conditions of imaging produce contrast inversion, due to image distortion induced by charged surfaces, and also carry information on the polarization direction in the domains. Finally, we show that the intensity profile at domain walls provides experimental evidence for a local stray, lateral electric field.

Many applications using ferroelectric materials depend on bulk properties, for example, photonic^1^, piezoelectric^2^ or magnetoelectric devices^3^. However, in electronics and catalysis the surface is often more important. The polar discontinuity at the bulk-terminated surface gives rise to a net fixed surface charge defined by 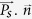, where 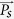 is the spontaneous polarization and 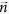 is the surface normal. This charge, and the different mechanisms by which it may be screened, can play a crucial role in the ferroelectric stability of thin films^4^,^5^, in surface reactivity^6^ and in the interface chemistry and electronic structure of, for example, ferroelectric capacitors^7^ or tunnel junctions^8^. Domain walls can also give rise to specific surface electronic and chemical properties. Eliseev *et al*. performed an in-depth theoretical study concerning the local polarization, elastic fields and charge accumulation at the intersection of domain walls and ferroelastic surfaces^9^. The influence of the surface on domain wall widths in ferroelectrics has also been investigated^10^.

Given the typical thickness of ferroelectric domain walls (1 to 10 nm), the number of techniques that possess a sufficient spatial resolution to study them remains limited. Optical techniques, such as Raman micro-spectroscopy^11^,^12^,^13^, second-harmonic-generation^14^ or defect luminescence microscopy^15^,^16^ provide promising results on domain walls although they are diffraction-limited. Most experiments use scanning probe microscopy (SPM)^17^ or transmission-electron-microscopy (TEM)^18^,^19^,^20^. However, both techniques have drawbacks: tip-surface interactions can influence the results of SPM^21^, and TEM requires a complex sample preparation which can influence the sample state. Furthermore, while TEM offers atomic resolution it cannot provide mesoscopic scale information, which is often the scale associated with ferroelectric domains.

Photoelectron emission microscopy (PEEM) also enables imaging of domain walls, as Schaab *et al*.^22^ demonstrated for ErMnO_3_. The spatial resolution was 50–100 nm, much larger than the wall thickness, but nevertheless allowed imaging of the wall. This is possible when the signature of the domain wall is quite distinct from that of the surrounding domains.

A more recently introduced technique for studying ferroelectric materials is Low Energy Electron Microscopy (LEEM). It provides full field, non-contact imaging of surface potentials. The technique exploits monochromatic, low energy electrons to map the surface potential with a spatial resolution typically better than 20 nm^23^, which is several times better than in PEEM. At very low kinetic energies, incident electrons are reflected by the surface potential. At higher energy, electrons penetrate the sample surface and are elastically backscattered. The transition between reflection and backscattering provides a direct measure of the surface potential, which can be related to the surface polarization charge of ferroelectric domains^5^,^24^,^25^ and to particular domain walls^26^. Attempts to determine the surface polarization charge from a single image have also been made^27^,^28^,^29^. However, a more detailed understanding of the relationship between the electron optics settings of the microscope, the intensity contrast and the measured surface potential maps is still required for in-depth studies of ferroelectric surfaces.

When probing surface charge with electrons, it is also important to bear in mind that, given the nanometric width of domain walls, local in-plane fields may be high and can easily dominate the image of the wall. This can make it impossible to resolve the physical wall since it will be masked by field effects extending over several tens of nanometers. However, these field effects can be very well characterized in LEEM and reveal signatures of the domain walls.

Here, we investigate domains and domain walls in the prototypical uniaxial ferroelectric, magnesium-doped lithium niobate (Mg:LNO (0001)). Magnesium doping was chosen since it induces inclined domain walls with non-zero fixed charge providing possible conduction paths when illuminated by UV light^30^. In a uniaxial ferroelectric only two domain orientations are permitted: labelled as P_up_ (polarization pointing upward to the surface) and P_down_ (polarization pointing downward into the bulk), providing a simple reference material for LEEM experiments. The aim is to clarify the contrast mechanisms arising in the presence of domain surface charges and domain walls. We show (i) that a shift in the MEM-LEEM transition is a reliable way to identify the polarization in domains, (ii) that out-of-focus imaging conditions produces intensity contrast inversions, and we experimentally evidence for the first time (iii) the presence of stray, lateral electric fields at domain walls.

## Sample Description and Experimental Methods

The sample was a 10 × 10 mm^2^ 5% Mg-doped single crystal of lithium niobate (0001) supplied by HC Photonics Corp. It was poled by the manufacturer with a regular pattern of ferroelectric domains: they cross the entire sample in the y-direction and are 9.3 μm wide. The sample was then chemically polished with HF. Immediately prior to insertion in the ultra-high vacuum LEEM system (base pressure 1 × 10^−9^ mbar), the surface was cleaned via 5 minutes ozone exposure to remove organic surface contamination.

The experiments were performed using a LEEM III microscope (Elmitec). The incident electron beam was emitted by a thermionic LaB_6_ electron gun at an accelerating voltage of *U*_*0*_ = −20 kV. The sample was at *U*_*0*_ + *SV*, where the Start Voltage (SV) defines the incident electron energy with respect to the sample surface. At low start voltages (SV → 0 V), the incident electrons are reflected by the potential above the surface, i.e. Mirror Electron Microscopy (MEM). At higher voltages (typically, 1 V < SV < 10 V) they penetrate the sample and backscattering occurs, i.e. Low Energy Electron Microscopy (LEEM). The position of the MEM-LEEM transition measures the surface potential. The reflected or backscattered electrons are reaccelerated into the objective lens and finally pass through the imaging column. A double multi-channel plate, screen and camera recorded the electron intensity as a function of SV in a typical field of view of a few tens of microns. An angle-limiting aperture in the back focal plane of the objective lens cut off highly deviated electrons and improves spatial resolution. A schematic of the setup is shown in Fig. 1 of ref. 23.

Since the band gap of lithium niobate is large (3.8 eV^31^), electrons penetrating the sample can easily induce charging, distorting the LEEM images. Heating the sample up to 300 °C during the measurement increases the electron mobility and avoids these charging effects. The Curie temperature of Mg:LNO is 1200 °C^32^, therefore the sample remains at all times in the ferroelectric phase. Furthermore, the surface potential remained similar for temperatures in the range 160–450 °C. All images were corrected for detector inhomogeneities by a flat-field acquired at SV = 10 V.

Piezo-response Force Microscopy (PFM) images were obtained using an Innova (Bruker) microscope, after the LEEM experiments. PFM image acquisition occurs via contact mode using a 4 V AC bias voltage applied to a Cr/Pt-coated cantilever with a spring constant of 40 N/m (Tap300E from BudgetSensors). High voltages were applied with a home-made setup using a Keithley 2410. Silver paste on the rear of the sample provides a back electrode.

## Results and Discussion

### Piezo-response Force Microscopy

**Figure 1:**
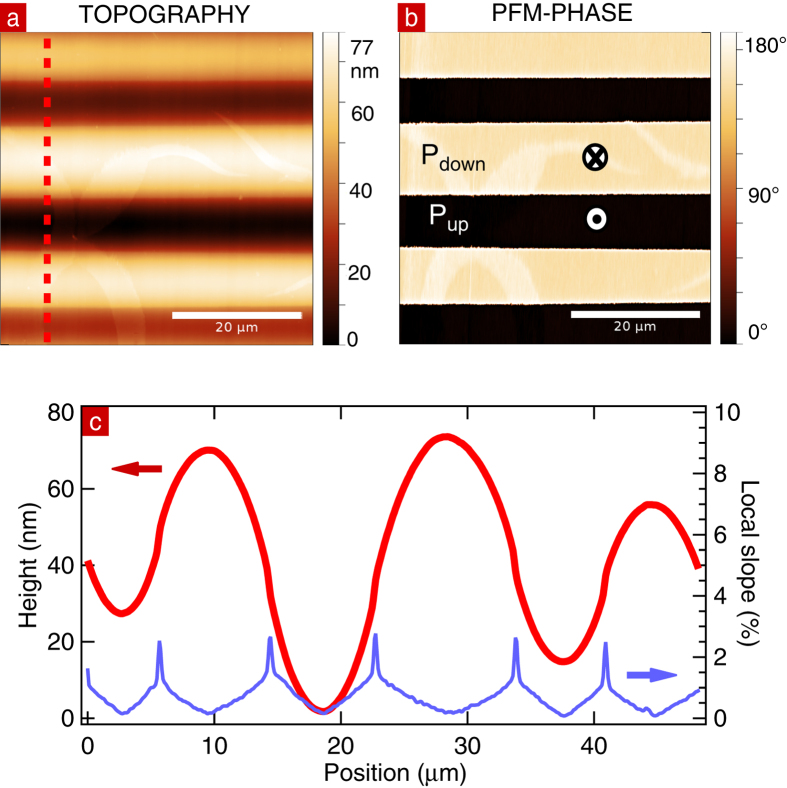
(**a**) PFM-topography image of the sample. (**b**) PFM-phase image of the same area. (**c**) Height profile along the red line on the topography image, and the corresponding local slope.

The domain structure of the sample was measured by PFM. Figure 1(a,b) shows the sample topography and PFM phase signal, respectively. The 180°-phase difference measured in PFM identifies domains of antiparallel polarization. Adjacent antiparallel ferroelectric domains have a height difference of 50 +/− 20 nm, due to the preferential etching of P_up_ surfaces. The profile extracted from the topography image, as indicated by the red line in Fig. 1(a) and displayed in Fig. 1(c), indicates smooth variation of height between domains with a slope always less than 2.25%. The roughness in the domains is below 0.3 nm. Neither the height variation nor the roughness are expected to contribute to contrast in the low energy electron images.

**Figure 2:**
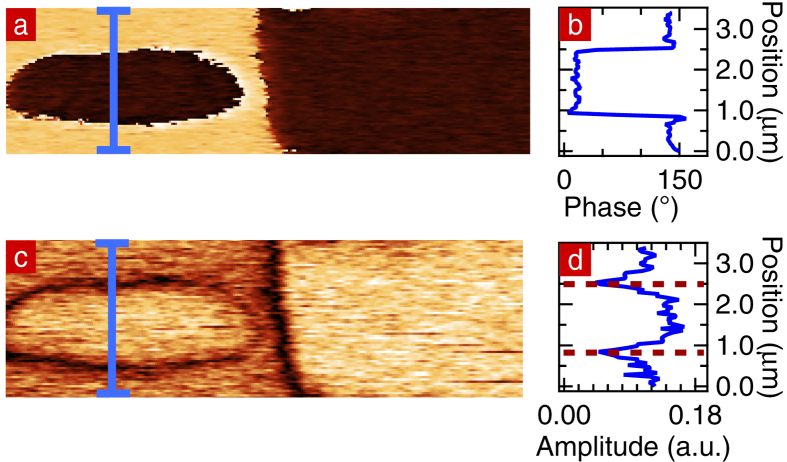
(**a**) PFM-phase image of the sample where −200 V was applied. (**b**) PFM-phase profile extracted along the blue line on the PFM-phase image. (**c**) PFM-amplitude image of the same area. (**d**) PFM-amplitude profile extracted along the blue line. The position of the domain walls are indicated by dotted lines.

In order to confirm the assignment of up/down polarization, we applied −200 V to the PFM tip to locally reverse the polarization of the surface. Figure 2(a) shows the PFM-phase image after poling. The dark domain does not change, whereas poling reverses part of the initially bright domain. Hence, in Fig. 2(a) the bright areas correspond to P_down_ and the darker areas to P_up_. Figure 2(b) plots the phase profile along P_down_ – P_up_ – P_down_, showing the characteristic PFM-phase of each domain. Figure 2(c) shows the out of plane component of the PFM-amplitude signal, proportional to the piezo-response. It can be seen that at each domain wall the amplitude is minimum, as expected for a 180° domain wall separating P_up_ and P_down_ domains.

### Low-Energy Electron Microscopy

Here we present the main results of the LEEM imaging of domains and domain walls. First, the variation of the MEM-LEEM transition allows mapping of surface potential variations. Then the use of MEM contrast to determine the sign of surface charge is discussed. Out-of-focus imaging conditions do not change the surface potential contrast between domains but do influence the apparent domain size. The intensity profile at domain walls is then related to the domain surface charge. Contrast inversion in the surface potential is observed in out-of-focus conditions contrary to the surface potential far from domain walls, providing an example of caustic effects in imaging.

### MEM-LEEM transition – Evaluation of the surface potential

**Figure 3:**
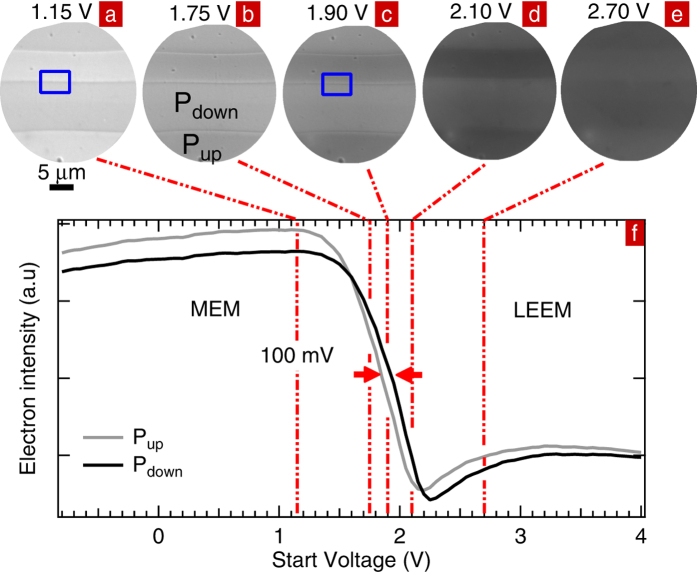
MEM images at start voltages of (**a**) 1.15 V and (**b**) 1.75 V. Same area observed in the region close to the MEM-LEEM transition at start voltage of (**c**) 1.90 V and in LEEM at start voltages of (**d**) 2.10 V and (**e**) 2.70 V. (**f**) Electron intensity curves extracted from P_up_ and P_down_ domains.

Figure 3(a,b) displays MEM images at start voltages of 1.15 V and 1.75 V. In Fig. 3(a), P_up_ and P_down_ domains appear as bright and dark stripes, respectively. Figure 3(d,e) displays the LEEM images in the same area, at start voltages of 2.10 V and 2.70 V, respectively. Figure 3(c) shows the electron image acquired in the region close to the MEM-LEEM transition. Both the image sharpness and the intensity decrease with respect to the MEM images but remain higher than the LEEM images. The electron images in LEEM are less sharp than in MEM. In LEEM, electrons penetrate the sample surface and although the LNO is Mg-doped and the measurements are carried out at 300 °C, some charging in the near surface region remains a possibility.

At 1.75 V, the contrast between domains reverses with respect to that at 1.15 V. Similarly, in LEEM at 2.70 V, the contrast is inverted with respect to that at 2.10 V. These contrast inversions are the signature of a shift in the MEM-LEEM transition between P_up_ and P_down_ domains, as can be seen from Fig. 3(f) which plots the electron intensity recorded in P_up_ and P_down_ domains as a function of SV. At low SV, the reflectivity is maximized; hence all electrons are reflected before reaching the sample surface. For SV above 1.20 V the intensity starts to decrease as incident electrons have enough energy to overcome the surface potential barrier and penetrate the sample surface. The domain for which the intensity decreases at lower start voltage has a lower surface potential, corresponding to the presence of positive surface charge, i.e. an upwards pointing polarization (P_up_)^5^,^24^. We can therefore identify the polarization of the domains, as shown in Fig. 3(b). The LEEM and PFM experiments occur in the same area and confirm the polarization deduced from the two experiments. At SV = 1.60 V, the intensity curves of the P_up_ and P_down_ domains cross giving rise to contrast inversion in the electron image. The MEM-LEEM transition for P_up_ (P_down_) is at 1.8 V (1.9 V). The shift in the MEM-LEEM transition between P_up_ and P_down_ domains, and therefore the difference in surface potential is ~100 meV.

The difference in surface potential can be compared with work functions and surface potential values obtained on lithium niobate by PEEM and Electrostatic Force Microscopy (EFM). The contrasts in work function and surface potential should be of the same order but with opposite sign. This statement has been confirmed experimentally on a BiFeO_3_ thin film where a work function difference of 300 mV was found in PEEM and a surface potential difference of 450 mV in LEEM^5^.

PEEM measurements on congruent lithium niobate^33^ have yielded a work function of ~4.6 V for P_down_ domains while that of P_up_ domains was greater than ~6.2 V, i.e. a work function difference above 1.6 V, one order of magnitude larger than our value. EFM measurements performed on stoichiometric lithium niobate in high vacuum were used to study the influence of external screening charges on the evolution of the surface potential^34^. The contrast was shown to linearly decrease–and even inverse–with time due the accumulation of screening charges. The same variations were observed with increasing temperature due to a reduction of polarization charges through the pyroelectric effect. If we extrapolate the measurements of Liu *et al*., at 300 °C, we expect a higher surface potential value in P_up_ domains with respect to P_down_ domains. We find the opposite.

The difficulty in comparing experimental values of surface potentials underlines the key role of screening, even in ultra-high vacuum experiments^35^, as previously shown in BaTiO_3_^25^. However, it is worth noting that a difference of ~100 mV between domains is of the same order of magnitude as observed in BiFeO_3_^5^, Pb(Zr,Ti)O_3_^24^ and BaTiO_3_^25^.

Such a shift is negligible compared to the 3.8 eV band gap and is unlikely to significantly change the domain wall conductivity. We recall that inclined domain walls in LiNbO_3_ require UV illumination to show higher conductivity^30^. In the work of Schaab *et al*., the purely in-plane polarization gave rise to highly charged head-to-head and tail-to-tail walls and showed a band bending of the order of 1 eV^22^. This could significantly change the conductivity compared with that of the surrounding domains, particularly as the band gap of ErMnO_3_ is only 2.5 eV^36^.

### Imaging of charged surface regions

At very low SV, well below the MEM-LEEM transition, reflectivity should be uniform across the field of view. Closer to the MEM-LEEM transition electrons are sensitive to positive or negative surface charge and hence to the ferroelectric domains. However, the surface charge creates stray fields which can deviate the electrons laterally^37^. Without converging lens between the sample (object) and the image, there is a simple correlation between the intensity variations observed on the screen and the physical or electrical topography. A hollow space in the surface (or a positively charged region) will focus the reflected electrons and therefore appear as a bright feature whereas a protrusion on the surface (or a negatively charged region) will scatter and defocus electrons^37^.

**Figure 4:**
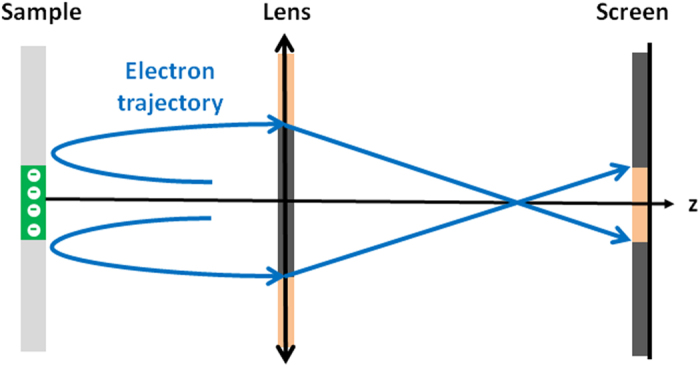
A schematic explaining the image formation of a charged region on the screen. The electron beam is deviated by a negatively charged region on the surface of the sample. In a direct image, the region appears as a large dark spot on the screen. In the presence of a converging lens, the electron beam becomes narrower on the screen and the negatively charge region appears as a small bright spot. Adapted from ref. 37.

In a LEEM, the presence of (several) convergent lenses in the imaging column modifies this. As can be deduced from Fig. 4, for an odd number of lenses in the imaging system, a negatively charged region gives rises to a bright image. For an even number of lenses the opposite is the case. In the present experiment, the imaging column is made of 5 lenses.

### Out-of-focus imaging of charged regions

**Figure 5:**
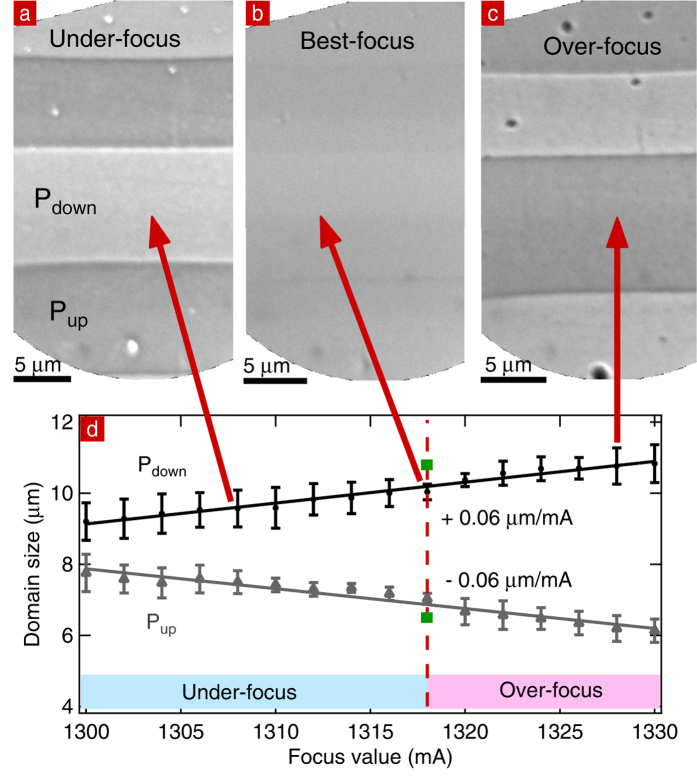
(**a**) Under-focus (**b**) best-focus and (**c**) over-focus images of the same area taken at 450 °C. (**d**) Evolution of the domain sizes with focus values expressed in coil current (mA): under-focus indicated by the light blue bar, best-focus indicated by the fuchsia dashed line, and over-focus images indicated by the light pink bar. Solid lines are linear fit of slope +0.06 μm/mA and −0.06 μm/mA. Green squares indicate the domain size as measured by PFM and reside on the best-focus line.

Usually, images are only taken at one focus value. Here, we will show that a systematic out-of-focus imaging can provide additional information, especially for large band gap samples. For this, we investigated the intensity contrast further by changing the focus value of the objective lens at a constant SV = 1.50 V. The results are presented in Fig. 5. In the LEEM the electron lens are magnetic, the lens setting is therefore measured in the coil current (mA). The higher the coil current, the stronger the focusing (and the shorter the lens focal length). Under-focus, P_down_ domains are bright while P_up_ domains are dark (Fig. 5(a)). If the focus value increases, P_down_ domains become darker and increase in size, while P_up_ domains become brighter and decrease in size (Fig. 5(b,c)). At over-focus, P_up_ domains are bright while P_down_ domains are dark (Fig. 5(c)).

Nepijko *et al*. demonstrated that if the surface potential difference between a negatively (positively) charged area and a substrate increases, the image size of the charged area increases (decreases) and its intensity decreases (increases)^37^. This is because the lateral field near the edge of the charged regions sweeps electrons outwards or inwards, respectively, making the pattern appear bigger or smaller in the image and as a consequence changing the image intensity. Varying the objective from under to over-focus is similar to varying the surface potential difference between regions with different surface charge: the higher the surface potential difference or the further out-of-focus, the stronger the distortion of the image. The focusing/defocusing is also responsible for a contrast inversion between P_up_ and P_down_ domains because the same number of electrons is imaged in a smaller or larger area, changing the intensity. Note that this does not represent a shift in the MEM-LEEM transition. Simulations show that over-focusing, for an odd number of converging lenses^37^, a negatively charged area (such as P_down_) is dark on the screen^38^,^39^. Figure 5(c) concurs with simulations.

Figure 5(d) shows the evolution of the imaged domain sizes with focusing expressed as current in the coil of the magnetic lens. The size of the field of view decreases with increasing focus values, we have therefore calibrated the scale for each focus value by using the distance between two defects. The domain size in the image changes linearly (straight lines in Fig. 5(c)) for the modest changes in focus of a few percent: it decreases for P_up_ and increases for P_down_. As a result, the intensity of P_down_ decreases with increasing domain size because the same number of electrons is now spread over a larger area in the image.

Since the variation of domain size depends on the surface charge, it can be used to identify the domain polarization. This method can therefore be useful to characterize samples with a large band gap where it is impossible to reach the MEM-LEEM transition because of charging even at low start voltage. It could also be used to identify the polarization of, for example, kinetic structures appearing across a phase transition using just two focus values.

However, a precise measurement of the domain size is not easy since it depends on the effective surface charge, which in turn is determined by the degree of screening, a priori not known. Thus the most reliable direct measurement of the domain size is by PFM whereas the surface charge is better revealed by LEEM. We measure a 10.8 μm (6.7 μm) width of the P_down_ (P_up_) domain of Fig. 5b in PFM and 10.0 μm (7.0 μm) in LEEM using best focus conditions. There is a maximum difference of 0.8 μm between both measurements: in LEEM, P_up_ and P_down_ sizes are under-estimated and over-estimated, respectively.

**Figure 6:**
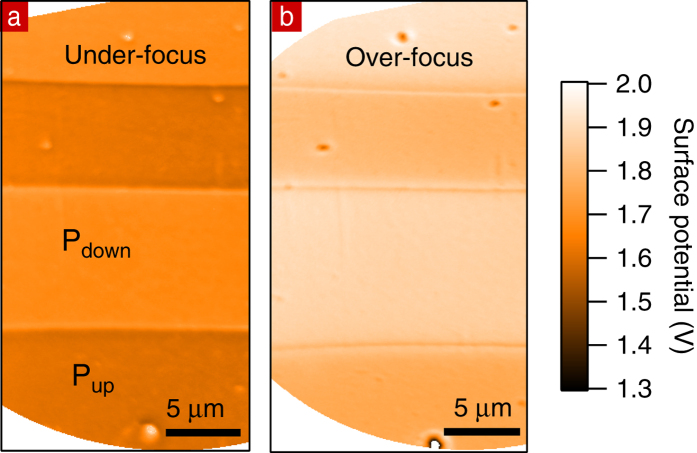
Surface potential maps for two focus values. (**a**) 1308 mA (**b**) 1321 mA.

Although the focus value changes the intensity contrast between domains, it does not influence the MEM-LEEM transition. This can be checked by recording image series as a function of SV in both under-focusing and over-focusing conditions. The value of the surface potential for each pixel is then determined by a fit of a complementary error function to the MEM-LEEM curve. The maps of the surface potential over the full field of view are shown in Fig. 6. The surface potential is always lower in P_up_ domains with respect to P_down_ by ~100 mV, irrespective of the focus value, although the absolute surface potential may shift. This makes the measurement of the surface potential difference between P_up_ and P_down_ robust against slight (here about 1%) defocusing.

### Domain walls

**Figure 7:**
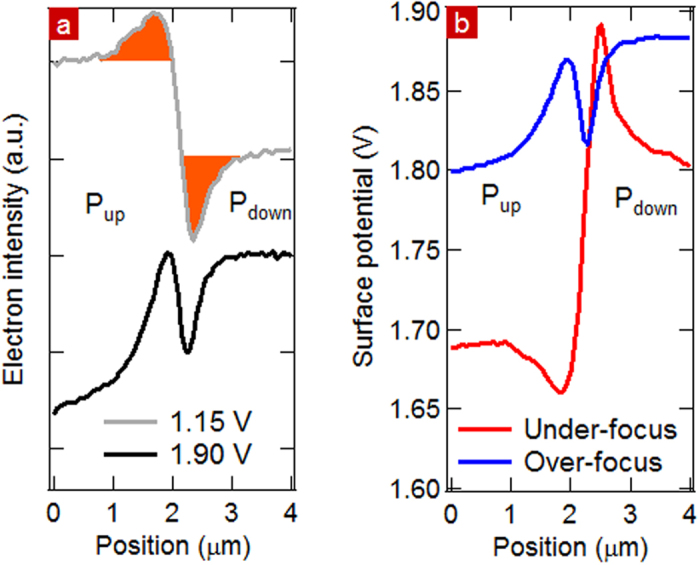
(**a**) Intensity profile in MEM (SV = 1.15 V) and LEEM (SV = 1.90 V). (**b**) Surface potential profile across a domain wall determined from the MEM-LEEM curves for over- and under-focus values.

In MEM, domain walls appear as successive bright and dark lines extending over 2 μm, two orders of magnitude higher than the spatial resolution. This clearly indicates that local field effects dominate in the electron image of the wall. The intensity profiles extracted across a domain wall within the blue rectangles on Fig. 3(a,c) are shown in Fig. 7(a). The maxima of the intensity correspond to the bright lines in the image while the minima correspond to the dark lines. The latter are on the P_down_ side of the domain wall while the bright lines are on the P_up_ side. In LEEM (Fig. 3(c)), the dark/bright order across the domain wall is the same as in MEM whereas the domain contrast inverts with SV.

**Figure 8:**
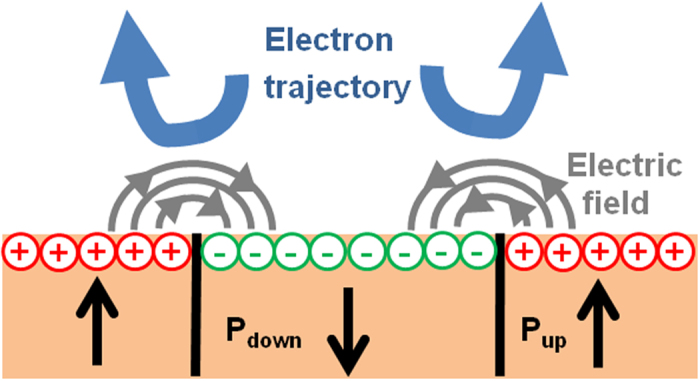
A schematic showing the lateral electric fields developing at the surface in the vicinity of domain walls and the deviation of the electron beam.

The difference in surface charge between domains gives rise to stray fields, located at domain walls, as shown in Fig. 8. If the magnitude of the stray lateral field is strong enough compared to that of the extractor (6.67 kV/mm), there is a significant lateral component which will deviate very low energy electrons from P_down_ towards P_up_ domains, as in Fig. 7(a). Local lateral fields alter less the higher energy electrons (>26 eV in this instance)^40^. As a result, near the vicinity of the domain wall, there are fewer electrons in P_down_ than in P_up_ domains, consistent with the successive changes of intensity where dark lines are near the P_down_ domains.

Figure 7(a) shows the deviated electron intensity via the areas below the “bright” peak and the “dark” peak in orange. They are approximately equal, consistent with a deviation of the electrons from the dark to the bright area in a direction parallel to the surface. Conservation of the total number of electrons indicates that at SV = 1.15 V both areas are still in the MEM regime. Similar intensity profiles occur for LEEM at 90° domain walls between in-plane polarized domains in BaTiO_3_^26^.

We have also tested the effect of under- and over-focusing on the domain wall contrast. Surface potential inversion occurs when changing from under-to over-focus conditions, as shown in Fig. 7(b) whereas the contrast between P_up_ and P_down_ domains is qualitatively unchanged far from the domain walls. The contrast inversion at the domain walls is an example of caustic effects due to the sharp variation in electron turning distance from the surface near the domain wall, changing the z motion of the electrons and therefore the image intensity as a function of SV. Varying from under to over-focusing gives rise to contrast inversion in the observed surface potential. The quantitative difference in surface potential between under and over-focusing far from the domain walls is due to the fact that we image charged regions and as discussed with respect to Fig. 5 the intensity changes as a function of focussing and hence the complementary error function returns a different value for the MEM-LEEM transition.

LEEM is sensitive to surface physical topography, since it gives rise to local electric fields that modify the electron trajectories. For example, height differences resulting from differential etching in ferroelectrics^41^ might give rise to intensity contrasts on the screen. Following Nepijko *et al*.^37^,^42^,^43^ both the aspect ratio and the absolute height difference are important in determining image intensity. Comparison between Fig. 1(a) and 3(a) already shows that the smooth variation of height between domains is not observed in the LEEM images where the domains appears as sharp rectangles with uniform intensity. This ensures that topography plays a negligible role in contrast formation in the particular case of chemically etched, Mg-doped LNO.

## Conclusions

We used Mirror and Low Energy Electron Microscopy to carry out a microscopic characterization of the domains and domain walls at the surface of Mg:LNO. We showed that the MEM-LEEM transition is a robust way to identify the polarization of ferroelectric domains. We demonstrated that out-of-focus settings can be used to determine the domain polarization and, more generally suggest that it is useful to study materials with a large band gap. For our sample, modest roughness and partial screening of the surface charge do not induce qualitative changes in the results.

We observe a strong in-plane electric field at domain walls arising from different surface charge states. The surface potential contrast at domain walls can be inverted by changing from under- to over-focusing conditions. We suggest that this is the signature of variation in electron turning distance due to rapidly varying local surface potential and is an example of caustic effects. Reliable characterization of the complex charge state and domain ordering at ferroelectric surfaces are therefore within the scope of low energy electron microscopy.

With respect to PEEM, LEEM has the advantage of using monochromatic electrons, thus at very low energy there is almost no secondary electron background, improving signal and resolution. However, the sensitivity to variations in local charge makes a direct measurement of microscopic domain sizes difficult. This is best done by a combination of LEEM and PFM.

## Additional Information

**How to cite this article**: Nataf, G. F. *et al*. Low energy electron imaging of domains and domain walls in magnesium-doped lithium niobate. *Sci. Rep.* **6**, 33098; doi: 10.1038/srep33098 (2016).
